# PD 0332991, a selective cyclin D kinase 4/6 inhibitor, sensitizes lung cancer cells to treatment with epidermal growth factor receptor tyrosine kinase inhibitors

**DOI:** 10.18632/oncotarget.13069

**Published:** 2016-11-04

**Authors:** Minghui Liu, Song Xu, Yuli Wang, Ying Li, Yongwen Li, Hongbing Zhang, Hongyu Liu, Jun Chen

**Affiliations:** ^1^ Department of Lung Cancer Surgery, Lung Cancer Institute, Tianjin Medical University General Hospital, 300052, Tianjin, China; ^2^ Laboratory of Lung Cancer Metastasis and Tumor Microenvironment, Tianjin Lung Cancer Institute, Tianjin Medical University General Hospital, 300052, Tianjin, China

**Keywords:** lung cancer, EGFR-TKIs, PD 0332991, gefitinib, drug resistance

## Abstract

Acquired resistance to epidermal growth factor receptor tyrosine kinase inhibitors (EGFR-TKIs) is a major challenge to targeted therapy for non-small cell lung cancer (NSCLC). We investigated whether a cyclin D kinase 4/6 (CDK4/6) inhibitor, PD 0332991, could reverse EGFR-TKI resistance in human lung cancer cells and explored the underlying mechanisms. We found that PD 0332991 potentiated gefitinib-induced growth inhibition in both EGFR-TKI-sensitive (PC-9) and EGFR-TKI-resistant (PC-9/AB2) cells by down-regulating proliferation and inducing apoptosis and G0/G1 cell cycle arrest. Tumor xenografts were then used to verify the effects of PD 0332991 *in vivo*. Mice treated with a combination of PD 0332991 and gefitinib had the fastest tumor regression and delayed relapse. Tumors from mice receiving the combination treatment exhibited down-regulated proliferation, up-regulated apoptosis, and less angiogenesis. Finally, lung adenocarcinoma patients with acquired resistance to EGFR-TKIs were given an exploratory treatment of PD 0332991. One patient with gefitinib resistance exhibited clinical remission after treatment with PD 0332991. These findings suggest PD 0332991 reverses acquired EGFR-TKI-resistance in NSCLC cells, and may provide a novel treatment strategy for NSLSC patients with EGFR-TKI resistance.

## INTRODUCTION

Lung cancer is one of the leading causes of cancer-related mortality in the world [1], and non-small-cell lung cancer (NSCLC) accounts for approximately 85% of all lung cancers [2]. Chemotherapy has traditionally played a central role in the treatment of patients with advanced NSCLC. However, the 5-year survival rate (less than 10%) is still very poor [3]. With the emergence of epidermal growth factor receptor tyrosine kinase inhibitors (EGFR-TKIs), the prognosis of adenocarcinoma patients with specific *EGFR* mutations has significantly improved [4]. Interestingly, *EGFR* mutations are more common in Asian female patients without a smoking history than in Caucasian patients. Adenocarcinoma patients with an *EGFR* mutation have a longer progression-free survival after treatment with the EGFR-TKI, gefitinib, as compared to standard chemotherapy [5, 6], making targeted therapy a hallmark of lung cancer treatment. Unfortunately, despite the success of EGFR-TKIs (such as gefitinib and erlotinib) in NSCLC patients, almost all cases eventually re-progress after a median of 10 months from the onset of treatment. Even the patients who initially exhibit a dramatic response will become resistant to EGFR-TKI treatment [2, 7–9]. Currently, this acquired resistance is the greatest challenge for EGFR-TKI treatment of lung cancer.

The mechanism of EGFR-TKI acquired resistance is likely multifactorial, but is not fully understood. For 40-50% of resistant lung cancers, the acquisition of a second mutation in *EGFR*, for example, the substitution of threonine with methionine at amino acid 790 (T790M) in exon 20, is a well-established mechanism [10, 11]. In addition, other mechanisms include *HER2* amplification [12, 13], *MET* amplification [14, 15], *PIK3CA* mutations [16, 17], *BRAF* mutation [18], *NF1* loss [19] and the activation of alternative signaling pathways [20]. Histologic changes, such as small cell lung cancer (SCLC) transformation or epithelial mesenchymal transition (EMT) have also been reported [21]. Despite the progress of mechanistic studies and emerging novel drugs, drug resistance is still a problem. The 3rd generation EGFR-TKI, AZD9291, is regarded as a breakthrough in the treatment of gefitinib- or erlotinib-resistant lung cancers. AZD9291 is an oral, irreversible, *EGFR* mutant-selective EGFR-TKI, which not only targets sensitive tumors (like L858R or exon 19 deletion) but also tumors with resistant T790M mutations [8]. Moreover, since other genes or signaling pathways are abnormally activated in TKI-resistant tumors, those targets are also exploited in the treatment of TKI resistance, although most of the drugs are still in preclinical or clinical trials [22]. However, all of these treatments still eventually lose efficacy and the disease progresses once again. Therefore, it is vital to find a solution to irreversibly treat TKI resistance.

Most cancer cells are killed after exposure to anticancer drugs. However, a small proportion of cells survives, escapes from the cell cycle, and enters into a quiescent stage (G0). In certain circumstances, the quiescent cancer cells will return into the cell cycle again from the G0 phase. This is called the “re-entry cell cycle” theory, which may also be applied as a theoretical mechanism of acquired resistance to EGFR-TKIs. Under this model, gefitinib or erotinib can kill most of the lung cancer cells harboring *EGFR* mutations, but the remaining cells are forced into G0 phase and escape from TKI damage. The exposure to EGFR-TKIs may block the EGFR pathway and force the tumor cells to acquire abnormal mutations or activation of oncogenes and/or alternative signaling pathways, resulting in tumor cell proliferation.

Therefore, in view of this theory, we propose that targeting the cell cycle might be a feasible method to reverse EGFR-TKI resistance. This treatment method can circumvent all the abnormally activated oncogenes or pathways and directly inhibit downstream factors, such as cell cycle-related proteins. In order to test our hypothesis, we conducted studies using PD 0332991, which is an orally active small molecule that potently and specifically inhibits cyclin D kinase 4/6 (CDK4/6) in a reversible manner. In preclinical studies and clinical trials, PD 0332991 had synergistic anti-tumor effects in combination with other drugs in breast carcinoma, multiple myeloma, and other tumors [25–29]. However, PD 0332991 has not been tested in EGFR-TKI-resistant lung cancers. Therefore, the purpose of present study was to investigate whether PD 0332991 can reverse EGFR-TKI-resistance in human lung cancer cells *in vitro* and *in vivo*, and to explore the underlying mechanisms.

## RESULTS

### PD 0332991 potentiated the growth inhibitory effect of gefitinib in both gefitinib-sensitive and resistant lung adenocarcinoma cell lines

**Figure 1:**
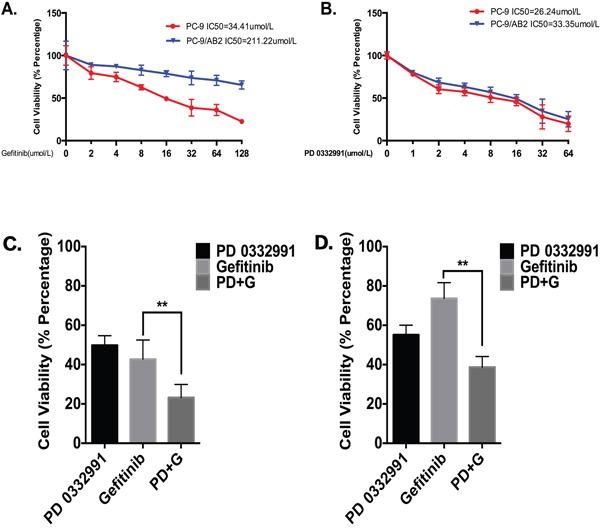
PD 0332991 enhances the growth inhibitory effects of gefitinib in PC-9 and PC-9/AB2 cell lines **A, B.** PC-9 and PC-9/AB2 cells were exposed to different doses of gefitinib (A) and PD 0332991 (B) for 24 hr to evaluate the IC50 of these two cell lines. MTT assay was used to evaluate cell viability. **C, D**. There was a synergistic interaction between PD 0332991 (8 μmol/L) and gefitinib (16 μmol/L) in PC-9 cells (C) and PC-9/AB2 cells (D). Cells were treated with various concentrations of gefitinib in combination with PD 0332991 for 24 hr, and cell viability was measured by MTT assay. The concentrations of PD 0332991 and gefitinib used in this study were from CompuSyn software (Combosyn, Inc.).

After exposure to gefitinib for 24 hr, PC-9 cells remained sensitive to treatment with an IC50 of 34.41 μmol/L, while PC-9/AB2 cells were less sensitive with an IC50 of 211.22 μmol/L (Figure 1A). PD 0332991 alone had no effect on cell viability (IC50 = 26.24 μmol/L for PC-9; IC50 = 33.35 μmol/L for PC-9/AB2; Figure 1B), although there may be other effects that were not tested. We next examined whether there was a synergistic effect of gefitinib and PD 0332991 on the viability of PC-9 and PC-9/AB2 cells (Supplementary Figure S1). As shown in Figure 1C, PD 0332991 at 8 μmol/L concentration potentiated the inhibitory effect of gefitinib at 16 μmol/L on PC-9 cells. Gefitinib at 16 μmol/L had no effect on PC-9/AB2 cell viability; however, the combination of PD 0332991 at 8 μmol/L and gefitinib at 16 μmol/L inhibited the growth of PC-9/AB2 cells (Figure 1D), suggesting that PD 0332991 has the potential to reverse EGFR-TKI resistance in NSCLC cells. Since PD 0332991 at 8 μmol/L and gefitinib at 16 μmol/L had a synergistic effect on the viability of PC-9/AB2 cells, this combination was used for subsequent *in vitro* studies.

### PD 0332991 enhanced the gefitinib-induced inhibition of cell proliferation, apoptosis, and G0/G1 phase arrest in lung adenocarcinoma cell lines

**Figure 2:**
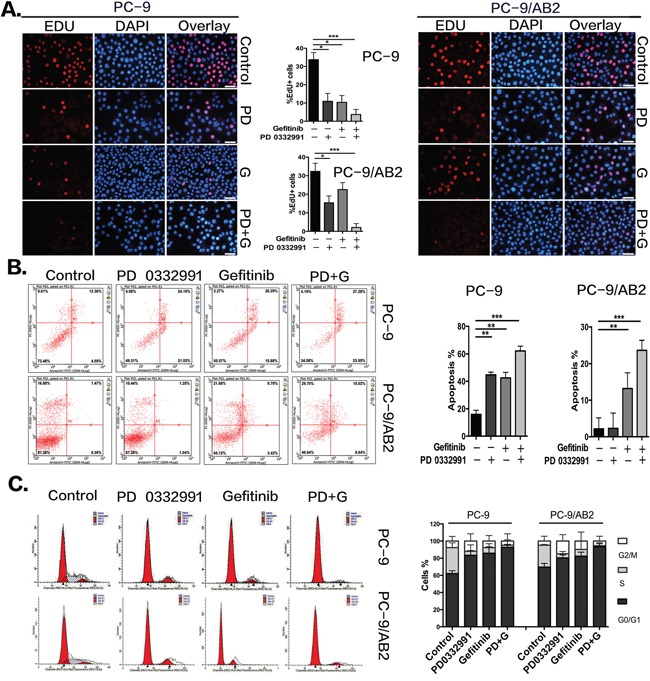
PD 0332991 enhances gefitinib-induced anti-proliferation, apoptosis and G1 phase arrest in gefitinib sensitive and resistant cells **A.** EdU assays were performed on the sensitive (PC-9) and resistant (PC-9/AB2) cells showing decreased levels of cell proliferation upon co-administration of PD 0332991 and gefitinib compared with either treatment alone. **B.** After 24 hr and exposure to PD 0332991 alone, gefitinib alone, or the combination of PD 0332991 and gefitinib, cells were prepared for AnnexinV/7AAD analysis by flow cytometry. **C.** PC-9 and PC-9/AB2 cells were incubated with gefitinib and/or PD 0332991 for 24 hr, stained with PI, and analyzed by flow cytometry. The cell cycle distribution shows the PD+G group had more G0/G1 phase arrest. Data are presented as mean ± SD (n=3 independent experiments). * P<0.05,** P<0.01,*** P<0.001, compared with the control group.

EdU staining was used to determine the effect of PD 0332991 on NSCLC cell proliferation. A single treatment of PD 0332991 (8 μmol/L) or gefinitib (16 μmol/L) inhibited PC-9 cell proliferation. The percentage of EdU-positive cells was 10.93% for the PD0332991 group, and 10.34% in the gefitinib group. The combination of PD 0332991 and gefitinib in PC-9 cells reduced EdU staining to 3.7% of cells. As expected, the gefitinib-resistant PC-9/AB2 cells were less sensitive to gefinitib (16 μmol/L). However, the percentage of EdU-positive PC-9/AB2 cells in the combination treatment group was reduced to 2.1%. These results indicate that PD 0332991 enhances the anti-proliferative effects of gefitinib (Figure 2A).

We next used AnnexinV/7AAD staining to assess cell apoptosis caused by PD 0332991 treatment. Compared to PC-9 cells, PC-9/AB2 cells had a much lower response to PD 0332991 or gefinitib, with an apoptosis rate of only 13.2% for the gefitinib group and almost no effect in the PD 0332991 group. However, the apoptosis rate of PC-9/AB2 cells increased to 23.66% with combined treatment of PD 0332991 and gefitinib. These results demonstrate that PD 0332991 can enhance gefitnib-induced apoptosis (Figure 2B).

Finally, we used PI staining to assess cell-cycle stages. A single treatment of gefitinib led to a 23.67% increase of G0/G1 arrest in PC-9 cells and a 12.75% increase in PC-9/AB2 cells. The combination of PD 0332991 and gefitinib led to a 30.6% increase in G0/G1 arrest in PC-9 cells and a 23.75% increase in PC-9/AB2 cells. These results indicate that the combination treatment of PD 0332991 and gefinitib induced a higher rate of G0/G1 arrest compared to treatments with PD 0332991 or gefinitib alone (Figure 2C).

### Microarray analysis

**Figure 3:**
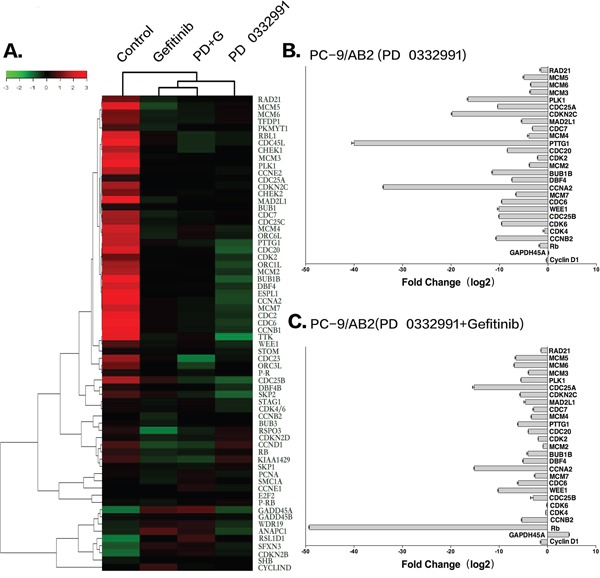
Microarray analysis of PC-9/AB2 cells treated with PD 0332991 and/or gefitinib for 24 hr **A.** The heat map associated with cell cycle genes. **B-C.** Real-time PCR of some cell cycle related genes to verify the results of microarray analysis.

To further explore the mechanism by which PD 0332991 sensitized TKI-resistant PC-9/AB2 cells to gefitinib, we performed microarray analysis to profile gene expression in PC-9/AB2 cells treated with PD 0332991 and a combination of PD 0332991 and gefitinib. There were 421 up-regulated and 1023 down-regulated genes in the PD 0332991 group (8 μmol/L) cells, while there were 1512 up-regulated and 1980 down-regulated genes in the combination treatment group (Supplementary Figure S2). We then examined the lists of genes for enrichment of Gene Ontology (GO) terms. A number of GO terms were enriched within both the PD 0332991 treated and the combination treatment PC-9/AB2 gene lists, including cell cycle checkpoint, G1/S transition of mitotic cell cycle, regulation of transcription involved in G1/S phase of mitotic cell cycle, and S phase of mitotic cell cycle. Kyoto encyclopedia of genes and genomes (KEGG) pathway analysis indicated that expression of genes in the DNA replication pathway, the cell cycle pathway, and the cell cycle-yeast pathways were enriched in both treatment groups (Figure 3A). To further confirm the microarray data, we performed real-time PCR for 27 cell cycle-related genes. As shown in (Figure 3B-3C), the expression of *CDK4*, *CDK6*, *CCNB2*, *CDC7*, and *CDK2* were all decreased after PD 0332991 treatment.

### PD 0332991 inhibits pRb phosphorylation of lung adenocarcinoma cell lines

**Figure 4:**
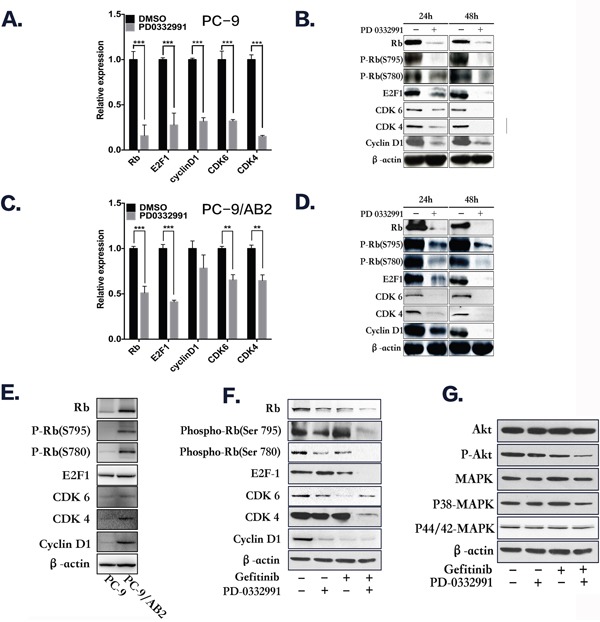
Western blot analysis and real-time PCR show that PD 0332991 and gefitinib down-regulate the expression of cell cycle-associated markers **A-B.** PC-9 cells treated with PD 0332991 alone for 24 and 48 hr. **C-D.** PC-9/AB2 cells treated with PD 0332991 alone for 24 and 48 hr. **E-G.** The expression of RB-E2Fs pathway proteins in PC-9 and PC-9/AB2 cells; The combination of PD 0332991 and gefitinib treatment in PC-9/AB2 cells for 24 hr resulted in changes in the RB-E2Fs and Akt/MAPK pathways.

The *RB1* gene was down-regulated in TKI-resistant PC-9/AB2 cells with combination treatment, therefore we hypothesized that inhibition of the pRb pathway by PD 0332991 contributes to the anti-cancer effects of gefitinib in TKI-resistant NSCLC cells. CDK4/6 complexes, along with CYCLIN D1, induce phosphorylation and inactivation of pRb, thereby allowing cell cycle progression. Of the 16 known phosphorylation sites on pRb, Ser780 and Ser795 are specifically phosphorylated by CDK4/6. Therefore, phosphorylation of pRb at these specific sites may be altered by the CDK4/6 inhibitor, PD 0332991. To test this hypothesis, we determined the effects of PD 0332991 on pRb and its phosphorylated state, as well as other cell cycle proteins using real time PCR and Western blots. There was a decrease in total pRb and phosphorylated pRb in both EGFR-TKI-sensitive and resistant NSCLC cells after treatment with PD 0332991 (8 μmol/L) for 24-48 hr (Figure 4A-4D). The expression of pRb, p-pRb(S780), p-pRb(S795), CDK4, CDK6, and CYCLIND1 were higher in PC-9/AB2 cells than PC-9 cells (Figure 4E). In addition, PD 0332991 treatment down-regulated the expression of E2F1, CDK4, CDK6 and CYCLIND1 in both cell lines.

The combination of PD 0332991 (8 μmol/L) and gefitinib (16 μmol/L) down-regulated phosphorylated pRb and E2F1 expression compared to either PD 0332991 or gefitinib alone in TKI-resistant PC-9/AB2 cells. Phosphorylated AKT and MAPK expression were also decreased with the combination treatment, indicating that AKT and MAPK are involved in the reversion of EGFR-TKI resistance by PD 0332991 (Figure 4F-4G). Because the effects of PD 0332991 could be the result of toxicity, we performed the experiments with various concentrations of PD 0332991 in PC-9/AB2 cells, and selected 1 μmol/L as the combined concentration with gefitnib (16 μmol/L). At this concentration we did not see any attenuation of CDK4/6 expression, and only a slightly attenuation of pRb expression, while P-pRb (S780), P-pRb (S795) and P-CDK6 (pTyr24) were all decreased (Supplementary Figure S3A). There were also synergistic effects with this lower dose as were seen with the other dose described above (Supplementary Figure S3B)

### Combination treatment with PD 0332991 and gefitinib inhibited the growth and relapse of human PC-9/AB2 tumor xenografts

**Figure 5:**
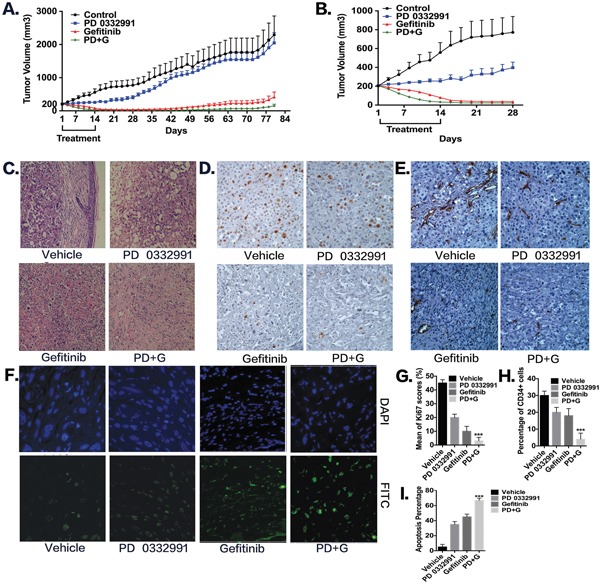
Combination of PD 0332991 and gefitinib treatment in PC-9/AB2 lung cancer xenografts Subcutaneous injections of 2 × 10^6^ PC-9/AB2 cells were performed with nude mice. When PC-9/AB2 tumors reached 200 mm^3^ in size, mice were randomized into four groups and administrated with the corresponding drugs as described in Materials and Methods. Tumor volumes were calculated using the formula; V=length^2^ x width^2^ x 0.5. Data, means ± SD; n=10 animals in each group. **A-B.** Tumor volume was measured and expressed as mean ± SD. **C-I.** Histologic sections were obtained from the mice harboring PC-9/AB2 tumors at day 10 after various treatments. HE staining (C), Ki-67 staining (D, G), CD34 staining (E, H), and TUNEL staining (F, I). * P<0.05, ** P<0.01,***P<0.001, compared with vehicle.

To further examine whether the combination of PD 0332991 and gefitinib altered tumor progression and relapse in gefitinib-resistant NSCLC cells *in vivo*, we used a PC-9/AB2 tumor xenograft mouse model. As shown in Figure 5A, a single treatment of PD 0332991 had an inhibitory effect on tumor growth, but the result was not statistically significant. However, administration of PD 0332991 (150 mg/kg) with gefitinib (100 mg/kg) inhibited tumor growth, showing tumor growth inhibition (168%; p < 0.001). All of the mice from the combination treatment group had a tumor volume < 30 mm^3^ after 14 days of treatment, and 20% (2/10) of the mice were completely cured without relapse. Importantly, mice treated with PD 0332991 plus gefitinib exhibited a much slow relapse pattern and increased survival times compared to gefitinib treatment alone (P+G 169.7 ± 4.62 days *vs* G 149.7 ± 6.87 days, *p*<0.05). Compared to the vehicle group, the PD 0332991 plus gefitinib group had a 40 day longer survival time. No severe side effects were observed in the PD 0332991 and/or gefitinib treatment groups. Interestingly, the mice treated with gefitinib alone also had inhibition of tumor growth. We speculate that this may be because the PC-9/AB2 cells have a different response to gefitinib *in vivo* than they do *in vitro*.

To explore the mechanism underlying tumor regression, we sacrificed a second set of identically treated mice on the 10^th^ day after treatment began, and tumor tissues were embedded and stained with HE, Ki67, TUNEL, and CD34. HE staining showed that the tumors from the PD 0332991 plus gefitinib group had more fibers and fewer blood vessels compared to the other three groups, indicating that they may have decreased metastatic capability (Figure 5C). Ki67 immunohistochemistry (Ki67-IHC) was used to assess cell proliferation. As shown in Figure 5D-5G, in the vehicle group, there was 45% positive staining, in the PD 0332991 group there was 21% positive staining, in the gefitinib group there was 12% positive staining, and the PD 0332991 plus gefitinib group, there was only 3% positive staining. These results indicated that PD 0332991 combined with gefitinib inhibited TKI-resistant cell proliferation *in vivo*. With TUNEL staining (Figure 5F-5I), we found that PD 0332991 plus gefitinib increased apoptosis rates as compared to other three groups (vehicle group: 5%, PD 0332991 group: 35%, gefitinib group: 45%, combination group: 67%). Finally, angiogenesis in tumor tissues was evaluated by CD34 immunohistochemistry (Figure 5E-5H). Angiogenesis was less abundant in the PD 0332991 plus gefitinib group compared to the other three groups (vehicle group: 30%, PD 0332991 group: 21%, gefitinib group: 18%, combination group: 4%; for CD34 positivity). These data indicate that the combination of PD 0332991 plus gefitinib induces apoptosis and inhibits proliferation and angiogenesis compared to PD 0332991 or gefitinib treatment alone in PC-9/AB2 tumor xenografts.

### Clinical analysis of pRb expression and case report

**Figure 6:**
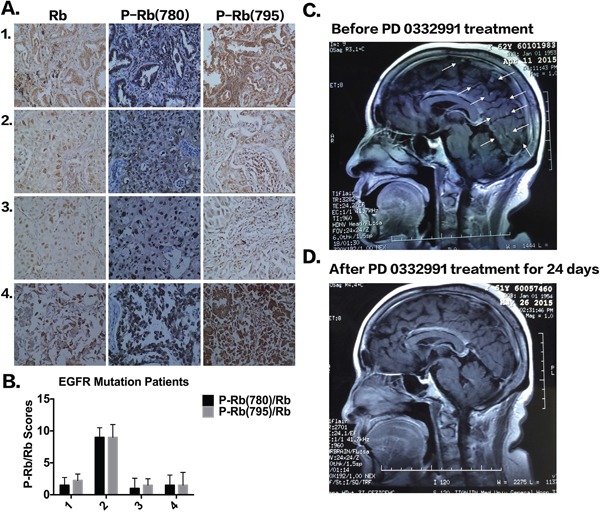
The expression of pRb and P-pRb in four patients with EGFR mutation **A.** IHC staining of pRb, P-pRb (S780) and P-pRb (S795) in four patients with *EGFR* mutations. **B.** P-pRb/pRb scores according to IHC staining. **C-D.** MRI examination of brain before and after PD 0332991 treatment in a patient with acquired EGFR-TKI resistance.

We next examined the expression of pRb in tumor tissues of NSCLC patients. As shown in Figure 6A-6B, the tumor tissues of NSCLC patients were all in phase IV, had *EGFR* mutations, and had high expression of phosphorylated pRb. The clinical characteristics of NSCLC patients are shown in Supplementary Table S2. Patient 2, who had a high p-pRb/pRb score (i.e. low pRb but extremely high phosphorylated pRb) developed gefitinib resistance after only 5 months of treatment, which was much less than the median time of 12 months (8,9,27). Three other patients with low or median p-pRb/pRb scores have not yet shown drug resistance after taking gefitinib for 1 year. However, due to the limited patient samples, it is impossible to determine whether phosphorylated pRb expression is correlated with the timing of EGFR-TKI resistance.

Three additional NSCLC patients who previously received EGFR-TKIs are receiving PD 0332991 treatment. Two patients had stable disease and one patient exhibited a partial response to PD 0332991 treatment. Figure 6 shows the patient, while the treatment pipeline is shown in Supplementary Table S3. The patient was a 63-year-old woman who presented with chronic cough for several months and was admitted in October, 2013. An enhanced chest computed tomography indicated a solitary mass in the upper lobe of the right lung. No distal metastasis was detected and a right upper lobectomy with systemic lymphadenectomy was performed. The pathological diagnosis was adenocarcinoma (pT2aN2M0, IIIA). Four cycles of postoperative chemotherapy were given with the regimen of pemetrex and cisplatin, followed by mediastinal radiotherapy of 5000Gy. The patient had bone and lung metastasis, and started to take Iressa (250 mg PO daily) and zoledronic acid (0.4 mg) infusion monthly. The metastatic tumors responded well and disease was controlled. However, brain metastasis was found, indicating that the disease had progressed and TKI resistance had occurred. The patient was given PD 0332991 (100 mg PO) treatment, while Iressa treatment continued. Three weeks after PD 0332991 treatment, MRI examination showed most of the brain metastasis had disappeared (Figure 6C-6D). Bone pain was also decreased. The patient is still taking PD 0332991 and under intensive care and follow-up. Although these findings are only from one patient, we believe that PD 0332991 may improve metastatic lung cancer patients’ quality of life and there should be further research on PD 0332991 in lung cancer patients.

## DISCUSSION

Lung cancer is a leading cause of cancer mortality worldwide [28]. Asian female patients without a history of smoking are more likely to have *EGFR* gene mutations in exon 19 or 21 compared to caucasians [29]. After a good initial response to EGFR-TKIs, of approximately 9-14 months, most patients will ultimately develop resistance to EGFR-TKIs. The mechanisms underlying acquired resistance are unclear. The primary mechanism identified is a secondary *EGFR* mutation, T790M, which occurs in ~60% of patients. More recently, an irreversible third generation EGFR-TKI, AZD9291, has emerged. However, acquired resistance still occurred despite an impressive initial response to AZD92921 in *EGFR* T790M+ patients, probably due to an additional C797S mutation [8]. Other mechanisms of resistance include *MET* amplification, *HER2* amplification, *EGFR* amplification and *PIK3CA* mutations [30]. Once patients appear resistant to EGFR-TKIs, re-biopsy and gene mutation detection is necessary. However, there are still many patients with EGFR-TKI drug resistance who do not have T790M or other secondary gene mutations. Recent studies have demonstrated that abnormal activation of alternative signaling pathways, including MET, ERBB2, AXL, and MAPK1, contribute to EGFR-TKI resistance [31–34]. It is important for future studies to investigate whether there is a common downstream mechanism of these alternative signaling pathways and secondary gene mutations.

Aberrant cell cycle control is a hallmark of cancer [35, 36]. Mutation of *EGFR* leads to the dysregulation of cell cycle control, ultimately facilitating proliferation that favoring tumorigenesis. EGFR-TKIs block the improper activation of the EGFR pathway. However, after a period of TKI treatment, NSCLC cells can reproduce via bypassing the EGFR pathway. The cancer cells acquire resistance to EGFR-TKIs and grow out of control with the activation of the cell cycle. Therefore, we hypothesize that cell cycle inhibition might be a good therapeutic target to reverse TKI resistance.

PD 0332991 is a potent and highly selective inhibitor of CDK4/6 and CYCLIND1 kinase activity. CDK4/6 phosphorylates pRb, a tumor suppressor and one of the most important proteins in the regulation of the cell cycle and cell proliferation. pRb expression is low in normal lung tissues, but the positive rate of pRb expression in NSCLCs is about 43.8% and is associated with a poor prognosis [37]. pRb forms a complex with E2F1 which is released upon phosphorylation of pRb, and is involved in regulating gene transcription and promoting cell proliferation. Consequently, inhibition of CDK4/6 by PD 0332991 completely suppresses pRb phosphorylation and exerts a potent antitumor effect. Previous studies have demonstrated that PD 0332991 has favorable anti-proliferative effects in breast cancer, ovarian cancer, myeloma, and glioblastoma cells *in vitro* [38, 39, 40]. In the present study we investigated the effect of PD 0332991 in EGFR-TKI-resistant lung cancer cells, both *in vitro* and *in vivo*.

We demonstrated that PD 0332991 caused EGFR-TKI-resistant NSCLC cells to overcome acquired resistance. A combination of PD 0332991 (8 μmol/L) and gefitinib (16 μmol/L) inhibited the viability of both EGFR-TKI-sensitive PC-9 cells and EGFR-TKI-resistant PC-9/AB2 cells, by down-regulating proliferation and inducing cell apoptosis and G0/G1 cell cycle arrest. Mice treated with PD 0332991 and gefitinib showed the fastest tumor regression and the slowest relapse pattern. Tumors from mice treated with the combination showed down-regulated proliferation, up-regulated apoptosis, and a reduced angiogenesis. Taken together, our data suggest that PD 0332991 enhances the effect of gefitinib-induced cell growth inhibition in both EGFR-TKI-sensitive as well as EGFR-TKI-resistant cells with no significant toxicity.

One important mechanism of EGFR-TKI acquired resistance is the activation of other signaling pathways bypassing EGFR and favoring cell cycle re-progression. After treatment with gefitinib, EGFR mutant NSCLC cells are arrested in the G0/G1 phase and become quiescent [41]. However, when alternative signaling pathways are activated, the cells in the G0 phase are able to re-enter the cell cycle and start to proliferate. In the cell cycle, the restriction point is G1-S phase, which is regulated by the CDK4/6-pRb pathway. Cyclins and the catalytic CDKs form active heterodimers to facilitate phosphorylation of pRb at Ser780 and Ser795 sites. After the NSCLC cells were treated with the CDK4/6 inhibitor, PD 0332991, CYCLIND1-CDK4/6 complexes could not be formed, and the downstream target, pRb, could not be phosphorylated and bind to the E2F1 transcriptional regulator, thus suppressing target gene transcription [42]. PD 0332991 blocked the CDK4/6-pRb-E2F1 pathway and promoted the EGFR-TKI-resistant cells to remain in G0 phase, suppressing growth. In breast cancer, PD 0332991 reduced tumor cell viability due to the loss of pRb function. However, we have not found other reports of PD 0332991 treatment in lung cancer.

In tissue samples, expression of phosphorylated pRb was high in tumors from NSCLC patients with *EGFR* mutations. However, due to the limited number of patient samples, it is difficult at this time to determine whether phosphorylated pRb expression is correlated with the time of EGFR-TKI resistance. To further translate our bench study into the clinic, three NSCLC patients were given PD 0332991. Two patients achieved stable disease and one patient had regression for brain metastasis. However, more patients with EGFR-TKI resistance need to be recruited to confirm the efficacy and side effects of PD 0332991.

In conclusion, we demonstrate for the first time that the CDK4/6 inhibitor PD 0332991 is a promising drug in the treatment of NSCLC patients with EGFR-TKI drug resistance by blocking pRb-mediated cell cycle progression. However, further studies are needed to predict a biomarker for PD 0332991 treatment and verify the effects of PD 0332991 in NSCLC patients with EGFR-TKI resistance. It is also worth investigating whether PD 0332991 can be used to combat resistance to other TKIs in NSCLC, such as AZD9291 or crizotinib.

## MATERIALS AND METHODS

### Drugs and cells

The parental NSCLC cell line, PC-9, and its drug-resistant sub-cloned cell line, PC-9/AB2, were provided by Prof. Zhou from Shanghai Pulmonary Hospital, Shanghai, China. PC-9 is a gefitinib-sensitive cell line that harbors a deletion of EGFR exon 19 (E746-A750del). PC-9/AB2 cells were established by long-term exposure of PC-9 cells to gefitinib and is resistant to gefitinib [23]. PC-9 cells were cultured in DMEM (GIBCO-BRL, Grand Island, NY) supplemented with 10% FBS (GIBCO-BRL, Grand Island, NY), in a humidified atmosphere of 5% CO_2_ at 37°C. Gefitinib, (N-(3-chloro-4-fluorophenyl) -7-methoxy -6-[3-(morpholin-4-yl) propoxy] quinazolin-4-amine) and PD 0332991were purchased from Selleck Chemicals, LLC (Houston, USA). Gefitinib was dissolved in DMSO and PC-9/AB2 cells were exposed at a concentration of 0.05 μmol/L.

### MTT assay

NSCLC proliferation was examined using a 3-(4, 5-dimethylthiazol-2-yl)-2, 5 diphenyltetrazolium bromide (MTT) assay. Cells (180 μl) in the exponential growth phase (3 × 10^3^ cells/well) were seeded into a 96-well plates, and 20 μl of gefitinib and/or PD 0332991 were added. After incubation at 37°C, 20 μl of MTT solution (5 mg/ml in PBS) was added to each well, and the plates were incubated for an additional 4 hr at 37°C. The plates were centrifuged at 200 x g for 5 min, medium was aspirated from each well, and 180 μl of DMSO was added to each well to dissolve the formazan. Optical density was measured at 490 nm with a SoftMax Pro analysis program interfaced with a SpectraMax M5 Microplate reader (Molecular Devices, Sunnyvale, CA, USA). Each experiment was carried out in 6 replicate wells for each concentration and in 3-4 independent experiments. The IC50-value was defined as the concentration needed for a 50% reduction in the absorbance calculated based on survival curves.

### 5-ethynyl-2'-deoxyuridine (EdU) staining

EdU staining detects the S-phase of the cell cycle by incorporating the nucleoside analog Uridine into newly synthesized DNA strands. A Cell-Light™ EdU stain kit was purchased from RiboBio (Guangzhou, China) and the staining was performed according the manufacturer's instructions. Briefly, cells were cultured with 50 μM EdU for 2 hr followed by two washes with PBS and fixation with 4% paraformaldehyde. After penetration by 0.5% Triton X-100 and washing with PBS, the proliferated cells were stained and analysis was performed with fluorescence microscopes using single interference filters set for red (Apollo), which stained EdU-labeled cells, or blue (Hoechst 33342), which stained all cell nuclei.

### Flow cytometry analysis of apoptotic cells

Cells (2 × 10^5^ cells/well) were seeded into 6-well plates and cultured for 24 hr. Gefitinib and/or PD 0332991 were added at various concentrations and cells were cultured for another 24 hr. Cells were stained using an Annexin V-FITC Apoptosis Analysis Kit (BD Bioscience, CA, USA) and analyzed with a FACSAria™ flow cytometer (BD Bioscience, CA, USA).

### Flow cytometry analysis of cell cycle

To determine the effects of gefitinib and/or PD 0332991 on cell cycle, 1.5 × 10^5^ cells/well were seeded in 6-well plates and incubated for 12 h. Cells were synchronized by starving them in serum-free DMEM for 24 h. Cells were then incubated with or without gefitinib and/or PD 0332991 for 24 h, trypsinized, and fixed in 70% ice-cold ethanol overnight. Cells were then treated with DNase-free ribonuclease (TAKARA, Shiga, Japan), stained with propidium iodide (PI) (Sigma–Aldrich, MO, USA), and analyzed using a FACSAria™ flow cytometer (BD Bioscience) and ModFit LT software (Topsham, ME, USA).

### Reverse transcription and quantitative real-time PCR

Total RNA was isolated from MSCs using Trizol (ThermoFisher Scientific, CA, USA) and an RNeasy Mini Kit (Qiagen), following the manufacturer's instructions. cDNA was synthesized using the M-MLV Reverse Transcriptase Kit (Promega, WI, USA) according to the manufacturer's protocol. Quantitative real-time PCR analysis was performed with ABI SYBR Green Master Mix (Thermofisher Scientific, CA, USA) in an ABI7500 Real-time PCR System according to the manufacturer's protocol. Each sample was run in triplicate for each gene. Transcript levels were normalized to the housekeeping gene phosphoglycerate kinase (PGK) and analyzed by the relative quantification 2-ΔΔCt method. All gene primers were obtained from SBS (Beijing, China). The primers are listed in Supplementary Table S1. To know other gene changes in PC-9/AB2 cells, we used Affymetrix GeneChip probe to test the gene changes in the RNA of PC-9/AB2 cell in four groups, Control, PD 0332991, Geftinib and PD+G. Affymetrix GeneChip Human Genome Array, which contains more than 54,000 probe sets to cover over 47,000 transcripts and variants, represent approximately 39,000 of the best characterized human genes, was used in microarray analysis. Hybridization, data capture, and analysis were performed by CapitalBio Corporation (Beijing, China), a service provider authorized by Affymetrix Inc. (Santa Clara, CA). Briefly, 100 ng of total RNA was used for cDNA synthesis, and produce biotin-tagged cRNA by Trizol method. 15μg fragmented cRNA, with contol oligo B2 and eukaryotic hybridization controls (bioB, bioC, bioD, cre) was hybridized to each GeneChip array at 45°C for 16 hours (Affymetrix GeneChip Hybridization Oven 640) according to manufacturer's instructions. After hybridization, the GeneChip arrays were washed, and then stained with streptavidin phycoerythrinonan (SAPE) with Affymetrix Fluidics Station 450 followed by scanning with the Affymetrix GeneChip Scanner 3000 7G.

### Antibodies and western blotting

Anti-E2F1, anti-CDK4, anti-CDK6, anti-CYCLIN-D antibodies were purchased from Santa Cruz Biotechnology (CA, USA). Phosphorylation site-specific anti-P-Ser780-pRb, anti-P-Ser795-pRb, anti-P-Ser473-AKT and anti-Phospho-p44/42-MAPK antibodies were from Cell Signaling Technology (Beverly, MA, USA). Anti-β-ACTIN was from Sigma–Aldrich (St. Louis, MO). Cells were homogenized in RIPA buffer (50 mM Tris-HCl, pH 7.4; 150 mM NaCl; 1% Nonidet P-40; 0.5% sodium deoxycholate; 0.1% SDS; 1 mM EDTA; 1 mM PMSF; 1 mg/ml Aprotinin), and proteins quantified using the bicinchoninic acid (BCA) protein assay kit (Pierce, IL, USA). A total of 10-50 μg proteins were fractionated on 10-12% gels using SDS-PAGE, transferred to nitrocellulose membranes (Amersham Biosciences, NJ, USA) under wet conditions, then immunoblotted with the appropriate antibodies.

### Tumor xenografts and survival analysis

BALB/c nude mice, 4-5 weeks old, were obtained from the Chinese Military Academy of Medical Sciences (Beijing, China). All mice were maintained under specific-pathogen free conditions and examined prior to the initiation of studies to ensure that they were healthy and had acclimated to the laboratory environment. PC-9/AB2 cells (2 × 10^6^) in 0.2 ml medium were injected subcutaneously into the right groin of each nude mouse. Tumor volume was determined by caliper measurements of tumor length (L) and width (W) according to the formula LW^2^/2. Mice were randomized into 4 groups when tumors reached 200 mm^3^ (10 mice per group): 1) Vehicle group, administered 0.5% methylcellulose, 400 (Wako Pure Chemical Industries, Ltd., Japan) and sodium lactate buffer by daily oral gavage; 2) PD 0332991 group, administered at 150 mg/kg/day PD 0332991 suspended in sodium lactate buffer (50 mmol/L, pH 4.0) and 0.5% methylcellulose, 400 by daily oral gavage; 3) Gefitinib group, administered 100 mg/kg/day gefitinib dissolved in 0.5% methylcellulose, 400 and sodium lactate buffer by daily oral gavage; and 4) PD 0332991 plus gefitinib group, administered 100 mg/kg/day gefitinib and 150 mg/kg/day PD0332991 simultaneously by daily oral gavage. All four groups received treatment for 14 days. Tumor size measurements were taken every other day.

### Immunohistochemistry

Serial (4 mm) sections from paraffin-embedded conventional tissues were deparaffinized in xylene and hydrated in a series of graded alcohols. Heat-induced antigen retrieval was carried out by microwave pretreatment in 5 mM Tris-HCl, pH 10.0, for 15 min. An overnight incubation at 4°C with primary antibodies (anti-CD34, at 1:500 dilution; anti-Ki67, at 1:500 dilution) was performed. Appropriate positive and negative controls were used for each experiment. The Kawai method was used to calculate a semi-quantitative score from 1 to 16 for staining of each tissue core. The number of positive cells was estimated and assigned a number score: 1 = <25%, 2 = 25%-50%, 3 = 50%-75%, and 4 = >75%. The intensity of staining was determined where 1 = none, 2 = weak, 3 = intermediate, and 4 = strong. The first and second scores were then multiplied resulting in a maximum staining score of 16 for any tissue core. Results were analyzed by Wilcoxon statistics, which correct for agreement by chance, and by percent agreement.

### TdT-mediated dUTP nick end labeling (TUNEL) assay

Serial (4 mm) sections from paraffin-embedded conventional tissues were deparaffinized in xylene and hydrated in a series of graded alcohols, followed by 3% hydrogen peroxide to block endogenous peroxidase activity and then digested with fresh Proteinase K37 for 1-15 min. The slides were stained using an In Situ Cell Death Detection Kit (Roche, IN, USA). Apoptotic cells with characteristic nuclear fragmentation were counted in four randomly chosen fields and calculated as the percentage of apoptotic cells.

### Statistical analysis

All data were analyzed by using the Statistical Package for Social Sciences (version 15.0.1, SPSS Inc., Chicago, Illinois, USA). Student's t-tests were used to identify the statistical significance between groups. ANOVAs were used for analysis of more than two groups of data in all experiments. Statistical significance: P<0.05.

## SUPPLEMENTARY MATERIALS FIGURES AND TABLES


