# Supplementary material for: PD 0332991, a selective cyclin D kinase 4/6 inhibitor, sensitizes lung cancer cells to treatment with epidermal growth factor receptor tyrosine kinase inhibitors
Source: Oncotarget. 2016 Nov 4;7(51):84951–64. doi: 10.18632/oncotarget.13069 (PMC5356711; doi:10.18632/oncotarget.13069)
Supplement: Supplementary file 1 [file oncotarget-07-84951-s001.pdf]

PD 0332991, a selective cyclin D kinase 4/6 inhibitor, sensitizes lung cancer cells to treatment with epidermal growth factor receptor tyrosine kinase inhibitors

SUPPLEMENTARY FIGURES AND TABLES

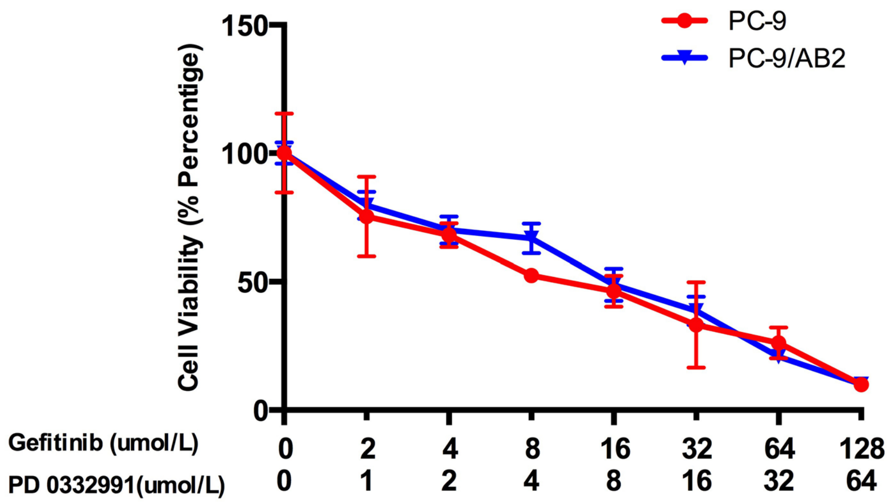

Supplementary Figure S1: Synergistic effect between PD 0332991 and gefitinib in PC-9 and PC-9/AB2 cells. Cells were treated with various concentrations of gefitinib in combination with PD 0332991for 24 hr, and cell viability was measured by MTT assay.

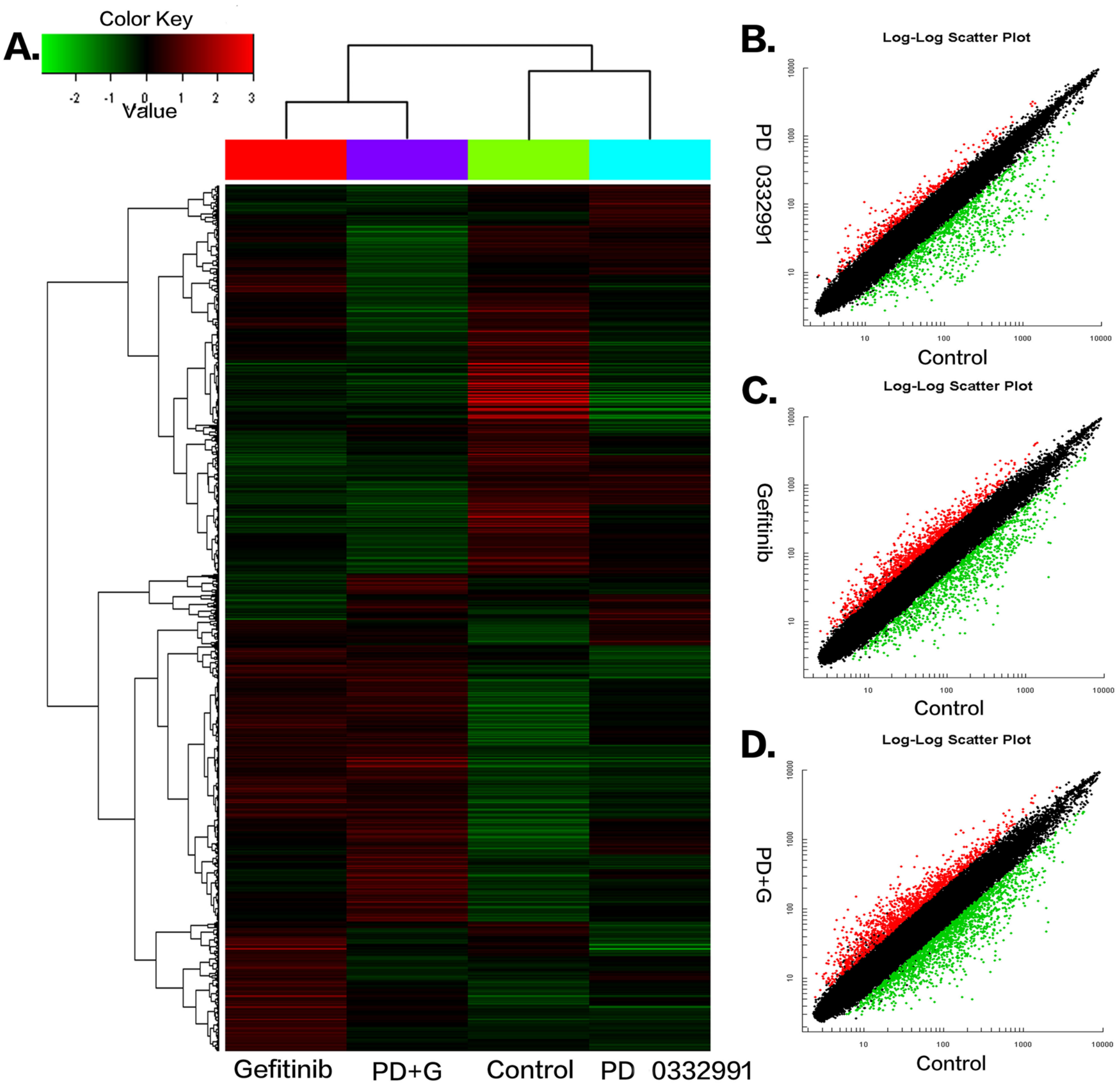

Supplementary Figure S2: Microarray analysis data in PC-9/AB2 cells treated with PD 0332991 and/or gefitinib. A. The heat map of alterations of gene expression in PC-9/AB2 treated with PD 0332991, gefitinib, or the combination of PD 0332991 and gefitinib. **B-D**. Scatter plot showing the comparison of PD 0332991, gefitinib, and PD+G group gene changes as compared with the control group.

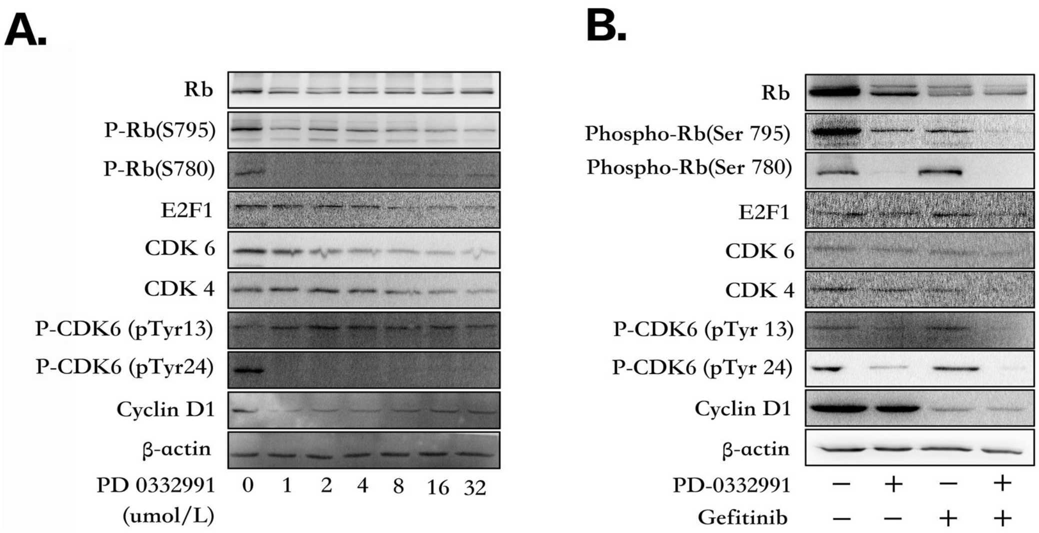

Supplementary Figure S3: Different doses of PD 0332991 and the effects of 1 µmol/L PD 0332991 combined with 16 µmol/L gefitinib in PC/AB2 cells. A. PC-9/AB2 cells were treated with different doses of PD 0332991 for 24 hr, which caused changes in the pRb-E2F1 pathway. **B.** The effects on the pRb-E2F1 pathway after 24 hr treatment with a combination of 1 µmol/L PD 0332991 and 16 µmol/L gefitinib in PC-9/AB2 cell lines.

Supplementary Table S1: The primer sequences for real-time PCR used to verify the results of gene expression changes found in the microarray analysis

Gene symbol	Forward primer	Reverse primer
RB1	ATCACAGCGATACAAACTTGGAG	AGCGCACGCCAATAAAGACA
E2F1	ACGTGACGTGTCAGGACCT	GATCGGGCCTTGTTTGCTCT
Cyclin-D1	GCTGGAGCCCGTGAAAAAGA	CTCCGCCTCTGGCATTTTG
MAD2L1	GGACTCACCTTGCTTGTAACTAC	GATCACTGAACGGATTTCATCCT
CDC25A	CTCCTCCGAGTCAACAGATTCA	CAACAGCTTCTGAGGTAGGGA
RAD21	AAGCCAAATACCTTCTTGCAGAC	CTGCTTCCCGATTTTCCTCAG
MCM3	GGCCTCCATTGATGCTACCTA	ACTTTGGGACGAACTAGAGAACA
MCM5	AGCATTCGTAGCCTGAAGTCG	CGGCACTGGATAGAGATGCG
CDC25B	GCATGGAGAGTCTCATTAGTGC	CTCCGCCTCCGCTTATTCT
MCM6	TCGGGCCTTGAAAACATTCGT	TGTGTCTGGTAGGCAGGTCTT
MCM2	ATGGCGGAATCATCGGAATCC	GGTGAGGGCATCAGTACGC
PLK1	CACCAGCACGTCGTAGGATTC	CCGTAGGTAGTATCGGGCCTC
CCNB2	TGCTCTGCAAAATCGAGGACA	GCCAATCCACTAGGATGGCA
CDC20	GCACAGTTCGCGTTCGAGA	CTGGATTTGCCAGGAGTTCGG
CCNA2	GGATGGTAGTTTTGAGTCACCAC	CACGAGGATAGCTCTCATACTGT
PTTG1	ACCCGTGTGGTTGCTAAGG	ACGTGGTGTTGAAACTTGAGAT
GADD45A	GAGAGCAGAAGACCGAAAGGA	CACAACACCACGTTATCGGG
BUB1B	AAATGACCCTCTGGATGTTTGG	GCATAAACGCCCTAATTTAAGCC
CDC6	ACCTATGCAACACTCCCCATT	TGGCTAGTTCTCTTTTGCTAGGA
CDKN2C	GGGGACCTAGAGCAACTTACT	CAGCGCAGTCCTTCCAAAT
DBF4	GGGCAAAAGAGTTGGTAGTGG	ACTTATCGCCATCTGTTTGGATT
CDK2	CCAGGAGTTACTTCTATGCCTGA	TTCATCCAGGGGAGGTACAAC
CDC7	AGTGCCTAACAGTGGCTGG	CACGGTGAACAATACCAAACTGA
MCM7	CCTACCAGCCGATCCAGTCT	CCTCCTGAGCGGTTGGTTT
MCM4	TGAACCTCTATACATGCAACGAC	CAGGGTAACGGTCAAAGAAGATT
WEE1	AGGGAATTTGATGTGCGACAG	CTTCAAGCTCATAATCACTGGCT

Supplementary Table S2: Clinical characteristics of patients with *EGFR* mutations

No.	Gender	Ages	EGFR Mutation	Stage	Information of EGFR-TKIs
1.	Female	51	Del19	IV	Gefitinib 12 months (No drug resistance)
2.	Male	52	Del19	IV	Gefitinib 14 months (Drug resistance)
3.	Male	26	Del19	IV	Gefitinib 5 months (No drug resistance)
4.	Female	51	L858R	IV	Gefitinib 15 months (No drug resistance)

Four patients with *EGFR* Mutations are all IV stage lung cancer. The gender of the patients is two female and two male. Three patients had Del19 mutations and one had an L858R mutation. They all had been treated with gefitinib for several months.

Supplementary Table S3: The treatment pipeline of the patient with EGFR-TKI acquired resistance using PD 0332991

Time	Treatment pipeline
2013-10-16	Right upper lobectomy, Adenocarcinoma (pT2N2M0, IIIA), EGFR 19 exon deletion
2013-12~2014-02	Pemetrex + Cisplatin, 4 cycles
2014-03	Mediastinal radiotherapy (5000Gy)
2014-08-12	Lung and bone metastasis, start to take Iressa (250mg PO daily) and zoledronic acid infusion monthly
2015-04-11	Brain metastasis, indicating drug resistance of Iressa
2015-05-02	Start to take PD-0332991 (100mg PO) +Iressa (250mg PO daily)
2015-05-26	Brain metastasis is barely detected.
